# The NF-κB RelA Transcription Factor Is Critical for Regulatory T Cell Activation and Stability

**DOI:** 10.3389/fimmu.2019.02487

**Published:** 2019-10-30

**Authors:** Emilie Ronin, Martina Lubrano di Ricco, Romain Vallion, Jordane Divoux, Ho-Keun Kwon, Sylvie Grégoire, Davi Collares, Angéline Rouers, Véronique Baud, Christophe Benoist, Benoit L. Salomon

**Affiliations:** ^1^Sorbonne Université, INSERM, CNRS, Centre d'Immunologie et des Maladies Infectieuses (CIMI-Paris), Paris, France; ^2^Division of Immunology, Department of Microbiology and Immunobiology, Harvard Medical School, Boston, MA, United States; ^3^Laboratoire NF-κB, Differentiation and Cancer, Université Paris Descartes, Sorbonne Paris Cité, Paris, France

**Keywords:** regulatory T cells, NF-κB, autoimmunity, stability, activation, relA

## Abstract

Regulatory T cells (Tregs) play a major role in immune homeostasis and in the prevention of autoimmune diseases. It has been shown that c-Rel is critical in Treg thymic differentiation, but little is known on the role of NF-κB on mature Treg biology. We thus generated mice with a specific knockout of RelA, a key member of NF-κB, in Tregs. These mice developed a severe autoimmune syndrome with multi-organ immune infiltration and high activation of lymphoid and myeloid cells. Phenotypic and transcriptomic analyses showed that RelA is critical in the acquisition of the effector Treg state independently of surrounding inflammatory environment. Unexpectedly, RelA-deficient Tregs also displayed reduced stability and cells that had lost Foxp3 produced inflammatory cytokines. Overall, we show that RelA is critical for Treg biology as it promotes both the generation of their effector phenotype and the maintenance of their identity.

## Introduction

CD4^+^ CD25^+^ Foxp3^+^ regulatory T cells (Tregs) play a critical role in immune homeostasis and in the prevention of autoimmune diseases by regulating immune responses (1). In humans and mice, it is well established that *forkhead box protein 3* (*Foxp3)* deficiency conducts to the development of an autoimmune syndrome leading to early death. Although *Foxp3* plays a critical role in the differentiation, suppressive function and stability of Tregs, other transcription factors (TFs), some of which interacting with Foxp3 in multi-molecular complexes, are also involved in different aspects of their biology. Some, such as c-Rel, are involved in Treg differentiation (2, 3). Others, such as NFAT, RunX1, BACH2, or Eos are critical to maintain their suppressive activity (4–7). Another group of TFs, including Blimp1, Myb, STAT3, Tbet, IRF4, Bcl6, or PPARg are involved in further differentiation of activated Tregs and in their capacity to suppress different types of immune responses (8–14). Finally, STAT5, TET, GATA3, p300/CBP, Blimp1, or Ezh2 have been shown to maintain Treg identity and stability by controlling Foxp3 transcription and epigenetics (15–20). Although it has been reported that NF-κB is able to bind to the regulatory sequence of *Foxp3* and to interact with a complex containing Foxp3 (2, 3, 21), its role in Treg biology needs to be further analyzed.

The NF-κB TFs consist of homo or heterodimeric molecules of NF-κB1 (p105/50), RelA (p65) and c-Rel subunits for the canonical pathway and of NF-κB2 (p100/52) and RelB subunits for the non-canonical pathway. It has been reported that c-Rel is essential for thymic Treg development by binding to the promoter sequence and the conserved non-coding sequence (CNS) 3 of *Foxp3* (2, 3, 22). The role of NF-κB in mature Treg biology has been addressed by knocking-out upstream activators of the pathway, such as IKKα and IKKß kinases. Mice with a conditional knockout (KO) in Tregs of either Ubc13, an E2 ubiquitin ligase activating IKKβ, or of IKKβ itself, develop a spontaneous autoimmune syndrome, associated with conversion of Tregs into effector-like T cells without Foxp3 loss or reduced Treg survival, respectively (23, 24). Mice with a conditional KO of IKKα in CD4^+^ T cells have a decreased proportion of Tregs in lymphoid organs, which seem to have a defective suppression and proliferation capacities *in vivo* (25). The specific role of RelA in Tregs, which is considered as the main factor of NF-κB members in conventional T cells (26), has been recently studied. By interacting with RelA and other TFs, such as Helios and p300, Foxp3 forms a multimolecular complex localized in active nuclear areas to act primary as a transcriptional activator (27). Mice with a conditional KO of RelA in Tregs develop a severe and early spontaneous autoimmune syndrome that is associated with a defect of effector Tregs (28–30). Here, we confirmed these latter findings and added further information on the nature of the disease with extensive description of lymphoid and myeloid cell activation in lymphoid and non-lymphoid tissues. Importantly, we revealed that RelA-deficient Tregs were unstable, lost Foxp3 expression and produced inflammatory cytokines, highlighting that RelA is also critical to maintain Treg stability and identity.

## Results

### Conditional Ablation of RelA in Tregs Leads to the Development of a Spontaneous Autoimmune Syndrome

**Figure 1:**
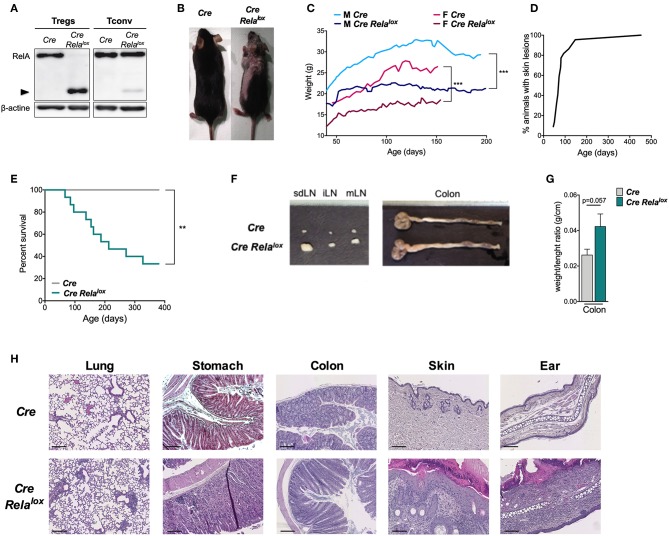
Mice with RelA deficient Tregs develop systemic inflammation. **(A)** Western blot analysis of RelA expression in Tregs and CD4^+^ conventional T cells (Tconv) isolated from *Foxp3*^*Cre*^ (*Cre*) and *Foxp3*^*Cre*^ *Rela*^*lox*^ (*Cre Rela*^*lox*^) mice. **(B)** Representative pictures of 12 week-old *Foxp3*^*Cre*^ and *Foxp3*^*Cre*^ *Rela*^*lox*^ mice. **(C)** Body weight monitoring of *Foxp3*^*Cre*^ and *Foxp3*^*Cre*^ *Rela*^*lox*^ males and females. **(D)** Percentages of *Foxp3*^*Cre*^ *Rela*^*lox*^ mice with skin lesions. **(E)** Survival monitoring of *Foxp3*^*Cre*^ *Rela*^*lox*^ mice. **(F)** Representative pictures from 20 mice of the LN and colon of 12 week-old *Foxp3*^*Cre*^ and *Foxp3*^*Cre*^ *Rela*^*lox*^ mice. **(G)** Weight/length ratio of colon of 12 week-old *Foxp3*^*Cre*^ and *Foxp3*^*Cre*^ *Rela*^*lox*^ mice. **(H)** Representative histology from 12 week-old mice of the lung, stomach, colon, skin, and ear of *Foxp3*^*Cre*^ and *Foxp3*^*Cre*^ *Rela*^*lox*^. Scale bars represent 200 μm (lung, *Foxp3*^*Cre*^ stomach, colon), 150 μm (*Foxp3*^*Cre*^ *Rela*^*lox*^ stomach), and 100 μm (skin, ear). Data are representative of independent experiments. Bars show the means and error bars represent SEM. For mouse and experiment numbers, see Supplementary Table 1. Statistical significance was determined using a log-rank (Mantel- Cox) test for the mouse survival data. The two-tailed unpaired non-parametric Mann–Whitney *U*-test was used. **p < 0.01, ***p < 0.001.

To assess the role of RelA in Treg biology, we generated *Foxp3*^*Cre*^ *Rela*^*lox*^ mice that have a specific deletion of RelA in Tregs by crossing mice expressing CRE in Tregs with mice expressing a *Rela* floxed allele. In these *Foxp3*^*Cre*^ *Rela*^*lox*^ mice, Tregs expressed a non-functional truncated form of RelA (Figure 1A), as expected using this floxed allele (31). From 5 to 10 weeks of age, *Foxp3*^*Cre*^ *Rela*^*lox*^ mice developed a spontaneous disease characterized by localized alopecia and skin lesions (epidermal hyperplasia, hyperparakeratosis, cystic hair), and reduced weight gain compared to *Foxp3*^*Cre*^ control mice (Figures 1B,C). This pathology had high penetrance and was severe since most of the animals had to be sacrificed for ethical reasons by 45 weeks of age (Figures 1D,E). At 10–12 weeks of age, *Foxp3*^*Cre*^ *Rela*^*lox*^ mice exhibited adenomegaly and macroscopic signs of mild colon inflammation (Figures 1F,G). Histological analyses showed moderate immune cell infiltration in the lung, stomach and colon and high level of immune cell infiltration in the skin (Figure 1H). The liver and small intestine were not or minimally infiltrated. Thus, mice with RelA-deficient Tregs developed a severe and systemic inflammatory syndrome.

**Figure 2:**
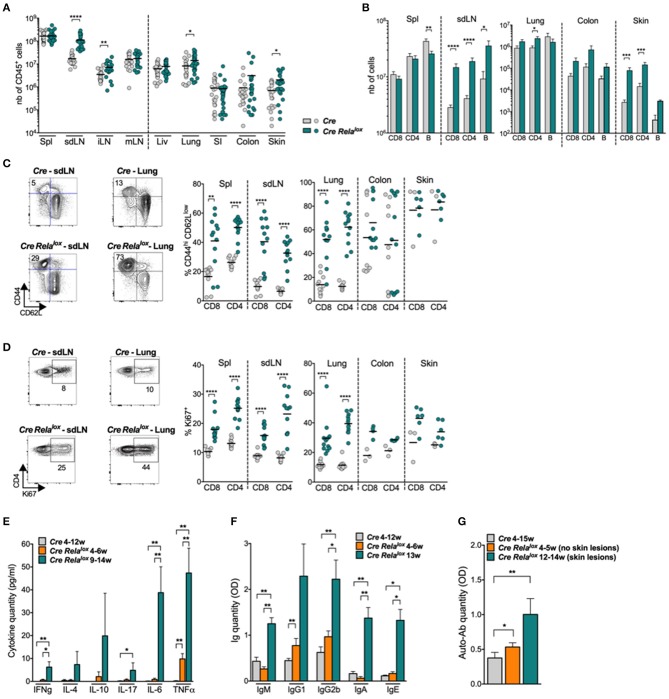
High activation of T and B lymphocytes in *Foxp3*^*Cre*^ *Rela*^*lox*^ mice. **(A,B)** Number of CD45^+^ **(A)**, CD8^+^, CD4^+^, and B cells **(B)** in the indicated organs (spl, spleen; liv, liver; SI, small intestine) of 12 week-old *Foxp3*^*Cre*^ (*Cre*) and *Foxp3*^*Cre*^ *Rela*^*lox*^ (*Cre Rela*^*lox*^) mice. **(C,D)** Representative dot plots and proportion of CD44^hi^ CD62L^low^ **(C)** and Ki67^+^ **(D)** among CD8^+^ and CD4^+^ Tconv in the indicated organs of 12 week-old *Foxp3*^*Cre*^ and *Foxp3*^*Cre*^ *Rela*^*lox*^ mice. **(E)** Cytokine quantification in the serum of 4–12 week-old *Foxp3*^*Cre*^, and 4–6 week-old and 9–14 week-old *Foxp3*^*Cre*^ *Rela*^*lox*^ mice. **(F)** Immunoglobulin quantification in the serum of 4–12 week-old *Foxp3*^*Cre*^ mice, and 4–6 week-old and 13 week-old *Foxp3*^*Cre*^ *Rela*^*lox*^ mice. **(G)** Anti-DNA antibody quantification in the serum of 4–15 week-old *Foxp3*^*Cre*^ mice, and 4–12 week-old and 12–14 week-old *Foxp3*^*Cre*^ *Rela*^*lox*^ mice. Each dot represents a mouse, lines and bars show the means of pooled independent experiments. Error bars represent SEM. For mouse and experiment numbers, see Supplementary Table 1. The two-tailed unpaired non-parametric Mann–Whitney *U*-test was used for data not following a normal distribution and the *t-*test was used for data following a normal distribution. *p < 0.05, **p < 0.01, ***p < 0.001, ****p < 0.0001.

We started the characterization of this syndrome by analyzing the lymphocyte compartment of 10–12 week-old *Foxp3*^*Cre*^ *Rela*^*lox*^ mice. Numbers of CD45^+^ leukocytes were highly increased in the skin draining lymph nodes (sdLN), the internal LN (iLN, corresponding to pancreatic and paraaortic LN) and the inflamed non-lymphoid tissues (lung and skin) but not in the spleen, mesenteric LN (mLN) or the non-inflamed non-lymphoid tissues (liver, small intestine) (Figure 2A). This leukocyte expansion was due to increased numbers of CD8^+^ and CD4^+^ T cells, B cells (Figure 2B and data not shown) and myeloid cells (see below). Moreover, the proportions of CD44^high^CD62L^low^, ICOS^+^, and Ki67^+^ activated/memory CD8^+^ and CD4^+^ conventional T cells were significantly increased in the spleen, sdLN, and lung of *Foxp3*^*Cre*^ *Rela*^*lox*^ mice compared to *Foxp3*^*Cre*^ control mice (Figures 2C,D and Supplementary Figure 1A). The same tendency was observed in the colon and skin, although this was not significant, probably because basal levels of activated cells were already high in *Foxp3*^*Cre*^ control mice. Interestingly, an increased proportion of activated/memory T cells was observed in the iLN and mLN as well as in the non-inflamed liver and small intestine, demonstrating a global systemic T cell activation in *Foxp3*^*Cre*^ *Rela*^*lox*^ mice (Supplementary Figure 1B). Systemic inflammation was confirmed by quantifying cytokines in the serum, where we observed highly increased levels of IFNγ, IL-4, IL-10, IL-17, IL-6, and TNFα (Figure 2E). Also, serum levels of IgM, IgG1, IgG2b, IgA, and IgE (Figure 2F) and of anti-DNA autoantibodies (Figure 2G) were increased in 12–14 week-old sick *Foxp3*^*Cre*^ *Rela*^*lox*^ mice compared to *Foxp3*^*Cre*^ control mice.

The systemic inflammation was further documented by analyzing myeloid cells, characterized as shown in Supplementary Figure 2A. Their numbers were strongly increased in the spleen and sdLN as well as in the inflamed non-lymphoid tissues, lung and skin, in *Foxp3*^*Cre*^ *Rela*^*lox*^ mice compared to controls (Supplementary Figure 2B). This increase of myeloid cells was due to an increase of neutrophils in all these tissues and of eosinophils and monocytes in the lymphoid organs and the skin (Supplementary Figure 2C). A similar trend was observed in the colon.

Only part of this inflammatory phenotype was observed in 4–6 week-old *Foxp3*^*Cre*^ *Rela*^*lox*^ mice. Increased numbers of whole CD45^+^ leukocytes were observed in sdLN and iLN but not yet in the lung and skin (Supplementary Figure 3A). A trend for higher proportion of activated/memory T cells, defined by expression of CD44, CD62L and Ki67, was observed in all analyzed lymphoid and non-lymphoid tissues of young mice (Supplementary Figure 3B). Finally, inflammatory cytokines, natural antibodies and anti-DNA antibodies were not or minimally increased in 4–6 week-old *Foxp3*^*Cre*^ *Rela*^*lox*^ compared to control mice (Figures 2E–G). In conclusion, *Foxp3*^*Cre*^ *Rela*^*lox*^ mice developed a severe systemic autoimmune syndrome, already uncovered at 4–6 weeks of age, followed, 1–3 months later, by massive activation of T cells, immune infiltration of several tissues and high rise of serum inflammatory cytokines, immunoglobulins, and auto-antibodies.

### Tregs of *Foxp3^*Cre*^ Rela^*lox*^* Mice Appear to Be Less Stable

**Figure 3:**
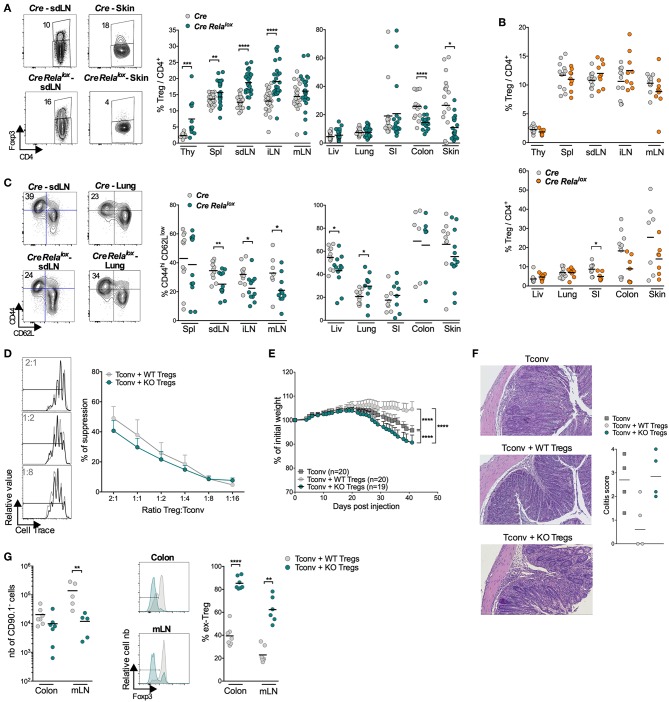
Tregs in *Foxp3*^*Cre*^ *Rela*^*lox*^ mice appear to be less stable. (A) Representative density plot and proportion of Tregs among the CD4^+^ T cells in the indicated organs (thy, thymus; spl, spleen; liv, liver; SI, small intestine) of 12 week-old *Foxp3*^*Cre*^ (*Cre*) and *Foxp3*^*Cre*^ *Rela*^*lox*^ (*Cre Rela*^*lox*^) mice. **(B)** Proportion of Tregs among CD4+ cells in 5 week-old *Foxp3*^*Cre*^ and *Foxp3*^*Cre*^ *Rela*^*lox*^ mice. **(C)** Representative density plots and proportions of CD44^hi^ CD62L^low^ among the Tregs of 12 week-old *Foxp3*^*Cre*^ and *Foxp3*^*Cre*^ *Rela*^*lox*^ mice. Each dot represents a mouse and lines show the means of pooled independent experiments. **(D)** *In vitro* suppressive activity of Treg cells from *Foxp3*^*Cre*^ (WT Tregs) and *Foxp3*^*Cre*^ *Rela*^*lox*^ (KO Tregs) 5–6 week-old mice. Representative data at 2:1, 1:2 and 1:8 (left) and different (right) Treg:Tconv ratios of independent experiments. **(E–G)** *In vivo* suppressive activity of Treg cells from *Foxp3*^*Cre*^ (WT Tregs, 6 week-old mice) and *Foxp3*^*Cre*^ *Rela*^*lox*^ (KO Tregs, 6 week-old mice) mice, determined in a colitis model stopped at 6 weeks for analyses. **(E)** Percentage of initial body weight pooled from independent experiments. Error bars represent SEM. **(F)** Representative histology of the colon and colitis scores. **(G)** Numbers of recovered Tregs (CD90.1^−^ cells), representative histograms and proportions of ex-Treg in the mLN and colon. Each dot represents a mouse and lines show the means of pooled independent experiments. For mouse and experiment numbers, see Supplementary Table 1. The two-tailed unpaired non-parametric Mann–Whitney *U* test was used for data not following a normal distribution and the *t-*test was used for data following a normal distribution. *p < 0.05, **p < 0.01, ***p < 0.001, ****p < 0.0001.

We then analyzed Treg homeostasis in 12 week-old *Foxp3*^*Cre*^ *Rela*^*lox*^ mice. Strikingly, Treg proportion was significantly increased in lymphoid organs, except in mLN, while it was decreased in the colon and skin and unchanged in the liver, lung and small intestine compared to *Foxp3*^*Cre*^ control mice (Figure 3A). Interestingly, in the small intestine, colon and skin of 5 week-old *Foxp3*^*Cre*^ *Rela*^*lox*^ mice, Treg proportion and number (except in the skin) seemed already decreased, when compared to 12 week-old *Foxp3*^*Cre*^ *Rela*^*lox*^ mice (Figure 3B; Supplementary Figure 4). The proportion of activated/memory CD44^hi^CD62L^low^ Tregs was decreased in all LN and the liver, and the same tendency was observed in the skin. However, their proportion was unchanged in the spleen, colon and small intestine and even increased in the lung (Figure 3C). Foxp3 and CD25 expressions were unchanged (data not shown).

The severe disease of *Foxp3*^*Cre*^ *Rela*^*lox*^ mice in the absence of major Treg quantitative defect suggests that Tregs may be dysfunctional. *In vitro* assays showed that RelA-deficient Tregs, purified from 5 to 6 week-old mice, were able to suppress proliferation of conventional T cells almost as efficiently as control Tregs (Figure 3D). To further analyze their function, we assessed their capacity to suppress colitis induced by effector T cells transferred into lymphopenic mice, measured by weight loss and histology. Surprisingly, not only RelA-deficient Tregs were unable to control colitis but the disease was even more severe compared to mice transferred with effector T cells alone (Figures 3E,F). This exacerbated colitis was not associated with increased number of cells from Tconv origin (CD90.1^+^ cells) or to their lower propensity to differentiate in peripheral Treg (pTregs) (Supplementary Figure 5). Instead, the severe colitis was rather due to the fact that most RelA-deficient Tregs lost Foxp3 expression in the colon and mLN, potentially differentiating in pathogenic effector T cells (Figure 3G). In conclusion, *Foxp3*^*Cre*^ *Rela*^*lox*^ mice had higher numbers of Tregs in lymphoid tissues (probably due to systemic inflammation) but lower numbers of Tregs in the colon and skin, which could be due to Treg instability and Foxp3 loss.

### RelA Deficiency Leads to a Defect of Effector Tregs at Steady State

**Figure 4:**
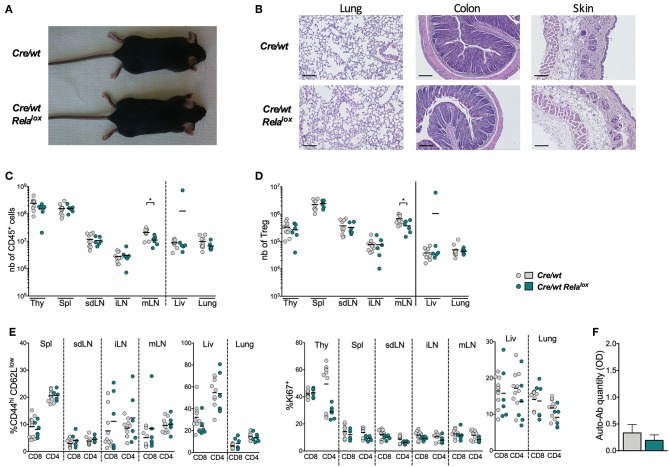
Heterozygous *Foxp3*^*Cre*/*wt*^ *Rela*^*lox*^ do not develop systemic inflammation. **(A)** Representative pictures of 8 week-old mice. **(B)** Representative histology of lung, colon and skin of 8 week-old. Scale bars represent 100 μm. Number of CD45^+^ **(C)**, of Tregs among CD4^+^ T cells **(D)**, and proportion of CD44^hi^CD62L^low^ and Ki67^+^ among CD8^+^ and CD4^+^ conventional T cells **(E)** in different tissues (thy, thymus; spl, spleen; liv, liver; SI, small intestine) of 8 week-old *Foxp3*^*Cre*/*wt*^ (*Cre/wt*) and *Foxp3*^*Cre*/*wt*^ *Rela*^*lox*^ (*Cre/wt Rela*^*lox*^) mice. Each dot represents a mouse and lines show the means of pooled independent experiments. **(F)** Anti-DNA auto-antibodies quantification in the serum of 8 week-old *Foxp3*^*Cre*/*wt*^ and *Foxp3*^*Cre*/*wt*^ *Rela*^*lox*^ mice. Bars show the means of pooled independent experiments and error bars represent SEM. For mouse and experiment numbers, see Supplementary Table 1. The two-tailed unpaired non-parametric Mann–Whitney *U* test was used *p < 0.05.

*Foxp3*^*Cre*^ *Rela*^*lox*^ mice developed systemic inflammation, which in return impacted on Treg biology. Thus, to assess the intrinsic role of RelA in Tregs at steady state, we generated *Foxp3*^*Cre*/*wt*^ *Rela*^*lox*^ heterozygous females, in which theoretically half of Tregs expressed RelA and the other half were RelA-deficient because of the localization of *Foxp3* locus in the X chromosome. We observed that these mice did not have any sign of disease and inflammation, as first noticed by macroscopic observations and the absence of cell infiltration in tissues (Figures 4A,B), which was most likely due to the presence of functional RelA-sufficient Tregs. This was further confirmed by analyzing the numbers of CD45^+^ leukocytes and Tregs that were similar in *Foxp3*^*Cre*/*wt*^ *Rela*^*lox*^ females and *Foxp3*^*Cre*/*wt*^ controls (Figures 4C,D). Moreover, the proportions of activated conventional T cells (Tconvs), defined by the expression of CD44, CD62L and Ki67, was identical between the two mouse types (Figure 4E). Finally, no increased level of anti-DNA auto-antibodies were detected in the serum of the *Foxp3*^*Cre*/*wt*^ *Rela*^*lox*^ females (Figure 4F). Thus, *Foxp3*^*Cre*/*wt*^ *Rela*^*lox*^ heterozygous females represent a proper model to study the intrinsic role of RelA in Tregs.

**Figure 5:**
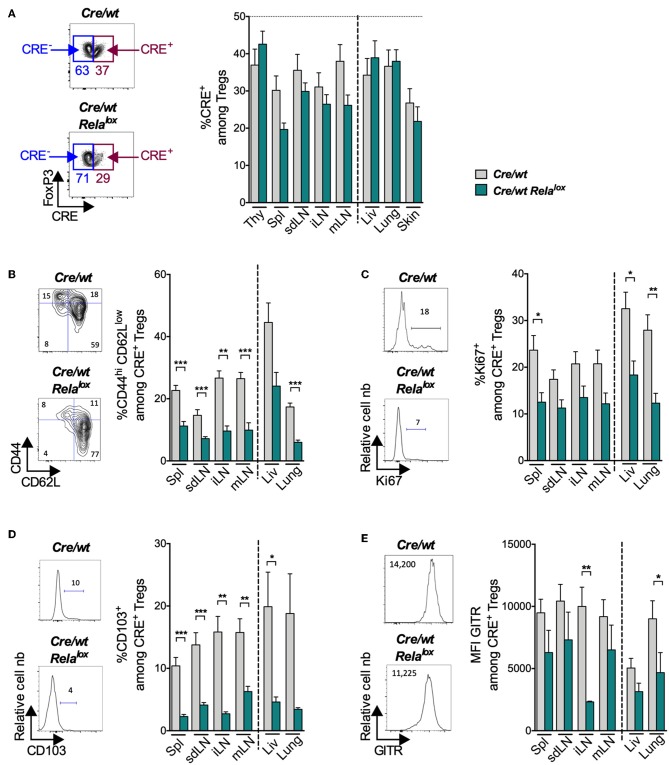
Reduced expression of activation markers in RelA-deficient Tregs at steady state. Analyses in the indicated organs (thy, thymus; spl, spleen; liv, liver) of 8 week-old *Foxp3*^*Cre*/*wt*^ (*Cre/wt* – gray bars) and *Foxp3*^*Cre*/*wt*^ *Rela*^*lox*^ (*Cre/wt Rela*^*lox*^–green bars) mice. **(A)** Representative density plots among CD4^+^ cells to define Tregs expressing CRE (CRE^+^) and percentages of CRE^+^ among total Tregs in sdLN. Representative density plots and proportions of CD44^hi^ CD62L^low^ **(B)**, Ki67^+^ **(C)**, CD103^+^ **(D)** and MFI of GITR **(E)** among CRE^+^ Tregs of sdLN. Bars show the means of pooled independent experiments and error bars represent SEM. For mouse and experiment numbers, see Supplementary Table 1. The two-tailed unpaired nonparametric Mann–Whitney *U*-test was used. *p < 0.05, **p < 0.01, ***p < 0.001.

In the *Foxp3*^*Cre*/*wt*^ control females, CRE-expressing Tregs (CRE^+^) were present in lower proportion compared to Tregs not expressing CRE (CRE^−^) (Figure 5A, gray bars). The same tendency was observed for the different molecules that we investigated (Figures 5B–E, gray bars), suggesting that the CRE transgene impacts on Treg biology in this competitive condition. Compared to these controls, the knockout of RelA did not modify significantly the proportion of Tregs (Figure 5A, green bars) nor the proportion of Tregs expressing ICOS, CTLA-4, Nrp1 or Helios (Supplementary Figure 6). However, the absence of RelA expression had a severe impact on Treg activation since the proportions of CD44^high^CD62L^low^, Ki67^+^, CD103^+^ and the expression level of GITR among CRE^+^ Tregs were strongly and systematically reduced (Figures 5B–E). In conclusion, RelA expression by Tregs appears critical for the acquisition of their effector phenotype at the steady state.

### RelA Plays an Important Role in Treg Activation

**Figure 6:**
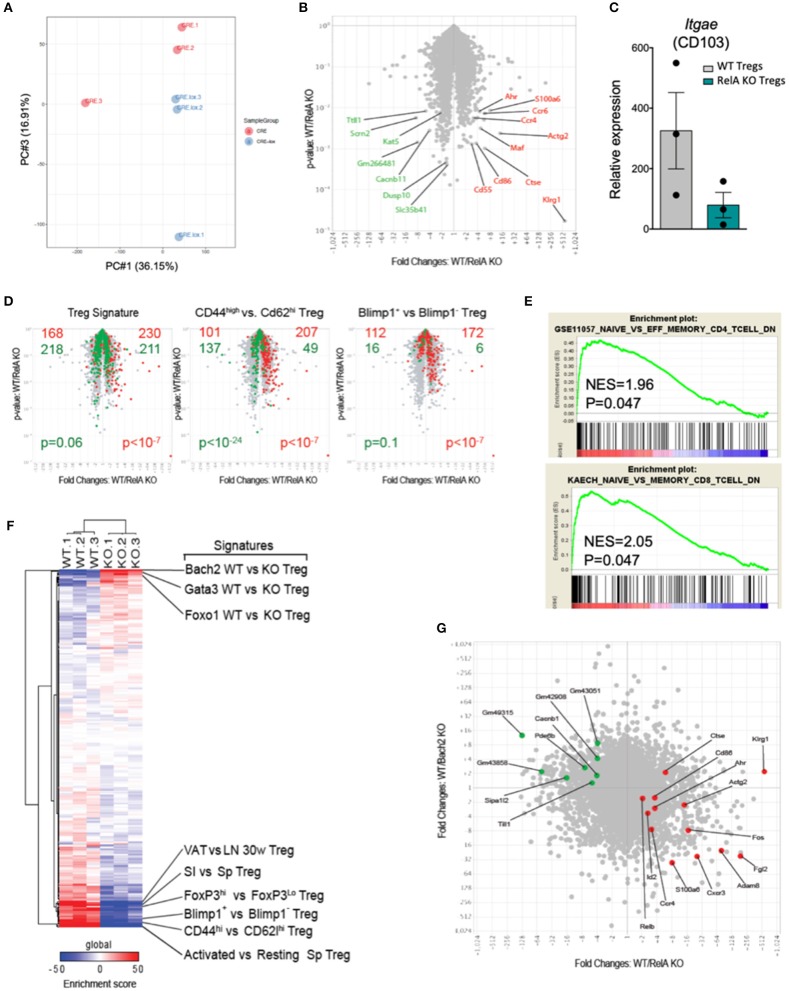
RelA-deficient Tregs have identity and activation defects. **(A)** PCA analysis of WT and RelA KO Tregs. **(B)** Volcano plot of WT vs. RelA KO Tregs. Red and green indicate transcripts up- and down-regulated, respectively, by WT Tregs cells. **(C)** Relative expression of *Itgae* (CD103) expressed in counts per million in WT and RelA KO Tregs. **(D)** WT vs. RelA KO Tregs (as in **A**) overlaid with various Tregs signatures. Red and green indicate genes up- and down-regulated, respectively, in each signature (chi-squared test for *p*-value). **(E)** GSEA plots of RelA-deficient Tregs compared with indicated set of genes up-regulated in effector memory CD4 (upper panel) and memory CD8 conventional T cells (lower panel) (32). **(F)** Heatmap for the enrichment score of each gene signature (VAT, visceral adipose tissue; LN, lymph nodes; SI, small intestine; Sp, spleen). **(G)** Fold change-fold change plot of WT vs. RelA KO Tregs (x-axis) and WT iTregs vs. WT Bach2 KO iTregs [y-axis, from published data (7)]. Red and green transcripts from **(A)**. For mouse and experiment numbers, see Supplementary Table 1.

To characterize more extensively the effects of the RelA deficiency on Tregs, we purified CRE-expressing Tregs from *Foxp3*^*Cre*/*wt*^ (WT) and *Foxp3*^*Cre*/*wt*^ *Rela*^*lox*^ (RelA KO) mice and profiled their transcriptomes by low-input RNAseq. Overall, transcriptome differences were modest (Figures 6A,B), with 180 differentially expressed genes at an arbitrary fold change cutoff of 2.0 (and false discovery rate < 0.05). The most biased transcript was *Klrg1*, as previously reported (28), but several other transcripts involved in Treg function and/or homing in the gut and skin showed a significant bias (e.g., *Ccr4, Ccr6, Maf, Ahr*, and *Itgae*) (Figures 6B,C). Gene ontology analysis did not reveal any evocative common pathway, so we projected various Treg-specific signatures onto the comparison of WT vs. RelA KO Tregs profiles (Figure 6D). RelA deficit modestly but significantly affected Treg identity as it reduced the canonical signature of genes differentially expressed in Tregs compared to Tconv cells (33) (Figure 6D, left). Moreover, consistent with the phenotype described above showing reduced proportion of activation markers in RelA-deficient Tregs in *Foxp3*^*Cre*/*wt*^ *Rela*^*lox*^ mice, a stronger bias was observed for signatures typical of activated Tregs [from comparison of CD44^hi^ vs. CD62L^hi^ Tregs, or from Blimp1- WT vs. KO Tregs (11)]. Indeed, RelA-deficient Tregs had a transcriptional signature analogous to CD62L^hi^ Tregs and Blimp1 KO Tregs, corresponding to resting-like Tregs (Figure 6D, middle and right). This effect was not unique to activated Treg signature, as GSEA analysis showed a strong bias of generic signatures of activated CD4^+^ or CD8^+^ Tconv cells (32) (Figure 6E). For further resolution, we cross-matched the RelA WT/KO difference to a curated series of 289 signatures that distinguish different sub-phenotypes of Tregs (34) (Figure 6F). The enrichment score of several gene sets characterizing activated or effector Tregs were decreased in RelA KO Tregs compared to WT Tregs (lower region of Figure 6F). Interestingly, however, RelA-deficient Tregs were enriched in several signatures resulting from the expression of TF with inhibitory roles in Tregs, and most markedly for Bach2 (upper region of Figure 6F). Indeed, the changes found here in response to RelA deficiency were largely anti-correlated with changes provoked by the absence of Bach2 in a previous report (7) (Figure 6G, *r* = −0.13 with *p* < 10^−15^ using a Pearson correlation). Overall, compared to WT Tregs, the transcriptomic signature of RelA-deficient Tregs confirmed their resting phenotype.

### RelA-Deficient Tregs Have a Defect of Stability

**Figure 7:**
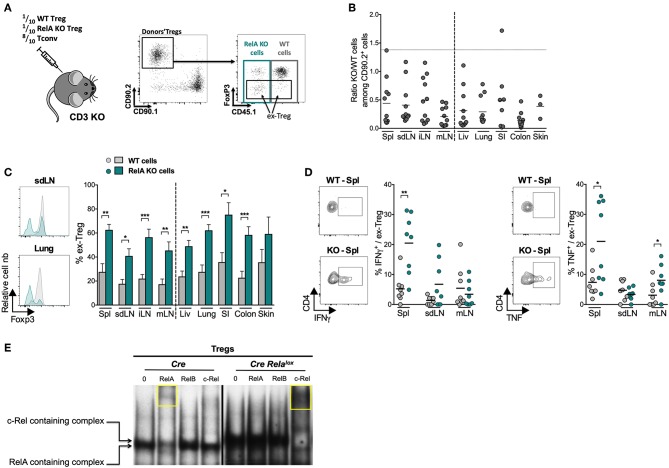
RelA-deficient Tregs are unstable and turn pathogenic. **(A–D)** Adoptive transfer of a 1:1:8 ratio of a mix of CRE-expressing Tregs from *Foxp3*^*Cre*/*wt*^ (CD45.1/2 CD90.2 WT Tregs), *Foxp3*^*Cre*/*wt*^ *Rela*^*lox*^ (CD45.2 CD90.2 RelA KO Tregs) mice and CD4^+^ conventional T cells (CD90.1 Tconv) into CD3 KO mice and analysis of donor cells 16 days later. **(A)** Experimental scheme and representative gating strategy from sdLN staining. **(B)** Ratio of RelA KO to WT Tregs in the indicated organs (spl, spleen; liv, liver; SI, small intestine) among CD90.2^+^ donor cells. The horizontal dot line represents the initial ratio (in the syringe). **(C)** Representative histograms and proportion of ex-Tregs from injected WT cells and RelA KO cells in different tissues. **(D)** Representative density plots and proportions of IFNγ^+^ and TNF^+^ cells among WT ex-Tregs and RelA KO ex-Tregs. Each dot represents a mouse, lines and bars show the means of pooled independent experiments. Error bars represent SEM. **(E)** EMSA combined with supershift assay analysis of NF-κB subunits activation in Tregs isolated from *Foxp3*^*Cre*^ (*Cre*) and *Foxp3*^*Cre*^ *Rela*^*lox*^ (*Cre Rela*^*lox*^) mice. The yellow squares point out the supershift of RelA or c-Rel containing complexes. The results are representative of independent experiments. For mouse and experiment numbers, see Supplementary Table 1. The two-tailed unpaired non-parametric Mann–Whitney *U*-test was used for data not following a normal distribution and the *t-*test was used for data following a normal distribution *p < 0.05, **p < 0.01, ***p < 0.001.

Our RNAseq data indicate an identity defect of RelA-deficient Tregs, which was first suggested in the colitis model (Figures 3E–G). However, one cannot conclude from this latter experiment that RelA plays an intrinsic role in Treg stability, owing to the very severe colitis developed by the mice injected with RelA-deficient Tregs. Indeed, increased instability of these latter could be well due to increased inflammation, and not RelA deficiency, since it is well established that different inflammatory factors precipitate Foxp3 loss (35). Thus, we further investigated whether RelA had any role in maintenance of Treg stability and identity by analyzing Foxp3 expression after co-transfer of RelA-sufficient and -deficient Tregs into the same mouse. Cells were purified from *Foxp3*^*Cre*/*wt*^ *Rela*^*lox*^ mice (*Foxp3*^*Cre*/*wt*^ for controls) and not from *Foxp3*^*Cre*^ *Rela*^*lox*^ mice, since systemic inflammation in these latter mice could modify Treg biology in addition to the impact of the RelA defect. Tregs were co-transferred in CD3 KO mice with Tconvs to sustain viability and expansion of injected Tregs (Figure 7A). Sixteen days after transfer, the proportions of RelA-deficient cells were much lower than the ones of RelA-sufficient cells (Figure 7B), particularly in the colon, a location subjected to high inflammation in this setting. Importantly, a large fraction of RelA-deficient Tregs lost Foxp3 expression, becoming so-called ex-Tregs, in all lymphoid and non-lymphoid tissues, compared to RelA-sufficient Tregs (Figure 7C). Moreover, RelA-deficient ex-Tregs expressed higher amounts of the pro-inflammatory cytokines IFNγ and TNFα, in the spleen and mLN, than their wildtype counterparts (Figure 7D).

To further explore the mechanism of Treg instability, and since it has been reported that c-Rel is involved in the differentiation of Th1 and Th17 cells (36, 37), we performed electrophoretic mobility shift assays **(**EMSA) combined with supershifts to assess the activation status of the different NF-κB subunits in Tregs of *Foxp3*^*Cre*^ and *Foxp3*^*Cre*^ *Rela*^*lox*^ mice (Figure 7E). In control Tregs, there was mainly an activation of RelA, rather than RelB or c-Rel. As expected, we did not observe this phenomenon in RelA-deficient Tregs, confirming that the truncated RelA protein was not functional. However, in Tregs of *Foxp3*^*Cre*^ *Rela*^*lox*^ mice there were much more activated NF-κB complexes, obviously due to the more activated phenotype of Tregs in these mice, which were mostly, if not only, constituted of c-Rel subunit. This massive c-Rel activation may be involved in Treg instability. In conclusion, our data show that lack of RelA activation strongly affect Treg stability leading to Foxp3 loss and increased differentiation of ex-Tregs, which may turn pathogenic through the production of inflammatory cytokines.

## Discussion

Here, we show that RelA plays a major role in Treg biology, both at steady state and during inflammation, since its specific deletion leads to the development of a spontaneous, severe, and systemic autoimmune syndrome.

The disease recapitulates some of the symptoms observed in Treg-deficient scurfy mice, although with a slower kinetics (1). As in scurfy mice, the skin and lymphoid organs are the most impaired tissues of *Foxp3*^*Cre*^ *Rela*^*lox*^, followed by the lung, stomach and colon and at lower extent the small intestine and liver. Also, we detected DNA auto-antibodies in the serum of our mice, as in scurfy mice (38, 39). We thus presume that *Foxp3*^*Cre*^ *Rela*^*lox*^ mice develop an autoimmune syndrome due to defective Tregs. Importantly, modification of the microbiota could play a major role in some tissue impairment such as the colon. Indeed, in *Foxp3*-deficient mice, colon damage becomes severe only after weaning, when microbial flora develops extensively (38). Our data suggest that this disease is initially due to a major activation defect of RelA-deficient Tregs. Indeed, in the *Foxp3*^*Cre*/*wt*^ *Rela*^*lox*^ non-inflamed mice, we observed reduced numbers of effector Tregs and suppressive molecules among the RelA-deficient Tregs. We thus speculate that in the *Foxp3*^*Cre*^ *Rela*^*lox*^ mice, and more specifically in tissues that are in contact with external environment and microbiota like the intestine and skin, effector T cells and myeloid cells become highly activated because of insufficient control by effector Tregs. Moreover, the decreased Treg proportion and number observed in those tissues in 5 week-old *Foxp3*^*Cre*^ *Rela*^*lox*^, potentially due to Treg instability and a decreased expression of gut and skin homing molecules (reduced mRNA levels of *Ccr4, Ccr6*) (40, 41), may exacerbate this phenomenon. Then, inflammatory factors may alter drastically stability of RelA-deficient Tregs most of them becoming pathogenic ex-Tregs, as we observed in the colitis model and cell co-transfer in lymphopenic mice experiments, precipitating local inflammation. The combination of reduced Treg number in the intestine and the skin, reduced Treg activation and the generation of pathogenic ex-Tregs may be the driving forces of the autoimmune syndrome of *Foxp3*^*Cre*^ *Rela*^*lox*^ mice.

Recent reports describe similar conditional KO mice developing a related autoimmune syndrome (28–30). They observed that *Foxp3*^*Cre*^ *Rela*^*lox*^ mice developed inflammation of the skin, stomach, lung and colon, massive activation of effector T cells and myeloid cells in lymphoid organs and high levels of inflammatory cytokines, immunoglobulins and anti-DNA in the serum. We confirmed these data and got deeper into the analysis of the disease since we showed that the effector T cells and myeloid cells were also drastically activated in multiple non-lymphoid organs. These data suggest a major defect of RelA-deficient Tregs. In addition, the injection of WT Tregs before 7 days of age was sufficient to stop the development of the pathology (data not shown). Surprisingly, we and others observed an increase of Treg proportion in lymphoid organs and *in vitro* assays did not reveal Treg suppressive defect. However, our extensive analysis enabled to point out a decrease of Treg proportion in the inflamed non-lymphoid tissues, such as the colon and skin. Our RNAseq analysis revealed a decreased expression of *Ccr4, Ccr6, Maf, Ahr*, and *Itgae* (encoding for CD103) which are involved in Treg function and/or homing in those tissues. Particularly, it has been shown that *Ahr* regulates the expression of *Ccr6* and *Itgae* and that *Ahr* deletion in Tregs leads to their decrease in the gut (42). As discussed above, this initial event may ignite the whole immune system, leading to widespread activation of the lymphoid and myeloid compartments and release of inflammatory cytokines that will boost global Treg activation and expansion, which remains insufficient to control the pathology.

Investigating initial events that led to disease could not be properly analyzed in *Foxp3*^*Cre*^ *Rela*^*lox*^ mice since inflammation has major impact on Treg migration, survival, activation, suppressive function or stability (35, 43), confounding the interpretation of what was due to inflammation or to the intrinsic RelA deficit. Using *Lck*^*Cre*^ *Rela*^*lox*^ mice, Messina et al. suggested that a major alteration of RelA-deficient Tregs was their defect to differentiate in effector Tregs (28). However, in this work, this defect was only partial, observed in LN and not in the spleen, and mostly analyzed in a quite irrelevant model since RelA was knockout in whole T cells. Vasanthakumar et al. showed a more global activation defect of RelA-deficient Tregs using *Foxp3*^*Cre*/*wt*^ *Rela*^*lox*^ mice or mixed bone marrow chimeric mice (29). We confirmed and completed these results by showing a downregulation of CD44, CD103, Ki67, and GITR not only in the lymphoid organs but also in the liver and lung of *Foxp3*^*Cre*/*wt*^ *Rela*^*lox*^ mice. Moreover, our transcriptomic analysis highlighted the major activation defect of RelA-deficient Tregs, since a strong bias was observed for signatures typical of activated Tregs. This reduced capacity of RelA-deficient Tregs to acquire an activation status could be due to an alteration of the proper function of the multimolecular complex normally containing Foxp3, p300, Helios, RelA, and other TFs acting as transcriptional activator (27).

What was more consistent and unexpected was the increased instability of RelA-deficient Tregs. This was first suggested in the colitis model, but more direct evidence came from studies where we compared RelA-sufficient and -deficient Tregs in the same environment after cell co-transfer in lymphopenic mice. We clearly showed that most RelA-deficient Tregs became ex-Tregs, contrary to control Tregs. Although with reduced intensity, increased instability of Rela-deficient Tregs was also observed in the absence of inflammation, as measured after transfer in lymphoreplete mice (data not shown). Moreover, we detected low amounts of the truncated RelA protein in the Tconvs of *Foxp3*^*Cre*^ *Rela*^*lox*^ mice, which may reveal the existence of ex-Tregs in these mice. Furthermore, we showed that these newly RelA-deficient ex-Tregs expressed inflammatory cytokines, suggesting that they could become pathogenic. This phenomenon may explain the increased severity observed in the colitis experiment and support our hypothesis that this ex-Tregs contribute to the pathology of *Foxp3*^*Cre*^ *Rela*^*lox*^ mice.

Foxp3 stability is controlled by histone and protein acetylation and by DNA methylation in the CNS 2 of *Foxp3* (44). RelA activity may impact on these epigenetic modulations by different ways. RelA interacts with CBP and p300 histone/protein acetyltransferases, which seems to be critical for the recruitment of CBP and p300 to their target promoter sites, as shown in fibroblasts (45). Because CBP and p300 promote *Foxp3* transcription, *Foxp3* stability at the level of CNS2 and prevent Foxp3 degradation (17, 46), RelA-deficient Tregs may have major instability. It has also been recently reported that RelA binds to genes involved in histone modification (29). Also, Foxp3 and RelA seem to cooperate to promote Foxp3 and CD25 expression by binding to their regulatory sequences (47, 48), which may favor Treg stability given the known role of IL-2 receptor signaling pathway in maintenance of Treg identity (16). Furthermore, Oh et al. recently reported that Foxp3 expression was down-regulated in Tregs of *Foxp3*^*Cre*^ *cRel*^*lox*^ mice and even more in the *Foxp3*^*Cre*^ *cRel*^*lox*^ *RelA*^*lox*^ mice, suggesting that RelA favors Foxp3 expression (30). Interestingly, we observed a dramatic increased binding of c-Rel to its target DNA sequence in Tregs of *Foxp3*^*Cre*^ *Rela*^*lox*^ mice. This phenomenon may hide the genuine role of RelA in Tregs and may further increase their conversion in pathogenic cells since c-Rel has been reported to be involved in Th1 and Th17 differentiation (36, 37).

Overall, our study further confirms the non-redundant role of RelA in Treg biology and reveals its new role in Treg stability. There are drugs targeting NF-κB subunits. Thus, it would be of strong interest to be able to target RelA in Tregs to propose new therapies triggering or inhibiting Tregs in autoimmune diseases or cancer, respectively. However, RelA has an important role in development and function of other immune cells (49–51). For instance, RelA is critical for CD4^+^ Tconv activation since its deletion prevent the development of autoimmunity in *Foxp3*^*Cre*^ *Rela*^*lox*^mice (28). Also, RelA is essential for differentiation and function of Th1, Th2, Th17, and Th9 cells (37, 52, 53). Therefore, a specific targeting of RelA in Tregs would be required.

## Experimental Procedures

### Mice

*Foxp3-CRE-IRES-YFP* (*Foxp3*^*Cre*^) (54), *RelA*^*flox*^ (31) and *Foxp3-IRES-GFP* (55) knock-in (*Foxp3*^*GFP*^) mice were kindly given by Prs. Alexander Rudensky, Falk Weih and Bernard Malissen, respectively. *CD3e*^*tm*1*Mal*^ (*CD3*^−/−^), *CD45.1, CD90.1*, and *RAG2*^−/−^ mice were obtained from the cryopreservation distribution typing and animal archiving department (Orléans, France). All mice were on a C57Bl/6 background. Mice were housed under specific pathogen-free conditions. All experimental protocols were approved by the local ethics committee “Comité d'éthique en expérimentation animal Charles Darwin N°5” under the number 02811.03 and are in compliance with European Union guidelines.

### Western Blot

Cells were lysed for 20 min on ice in extraction buffer (0.4 M NaCl, 25 mM Hepes pH 7.7, 1.5 mM MgCl2, 0.2 mM EDTA, 1%, NP4O, 20 mM glycerol phosphate, 0.2 mM Na3VO4, 10 mM PNPP, 2 mM DTT, 0.1 M PMSF). Whole cell extract was harvested after centrifuging the lysate for 10 min at 9,500 × g. 20 μg of whole cell extract were separated on 7.5% SDS–polyacrylamide gels and transferred to nitrocellulose membranes (GE Healthcare). Immunoblotting was performed with anti-RelA (C20) polyclonal antibodies (Santa Cruz Biotechnology) and anti-β-actin antibody (Sigma Aldrich) and visualized using the ECL Western blotting detection kit (Pierce).

### Histology

Organs were collected and fixed in PBS containing 4% formaldehyde for 48 h and then transferred in 70% ethanol. Five-micrometer paraffin-embedded sections were cut and stained with hematoxylin and eosin and then blindly analyzed.

### Cell Preparation From Tissues

For lymphoid tissues, cells were isolated by mechanical dilacerations. For non-lymphoid tissues, anesthetized mice were perfused intracardially with cold PBS. Small pieces of livers and lungs were digested in type IV collagenase (0.3 mg/ml) and DNase I (100 μg/ml) for 30 min at 37°C, followed by Percoll gradient (30–70%) separation. Small pieces of intestines, removed of their Peyer patches and epithelium, were digested in type IV collagenase (1 mg/ml) and DNase I (10 μg/ml) for 30 min at 37°C, followed by Percoll gradient (40–80%) separation. Small pieces of skin were digested in liberase DL (0.4 mg/ml), collagenase D (0.05 mg/ml) and DNase I (10 μg/ml) for 1 h at 37°C, followed by Percoll gradient (40–80%) separation.

### Antibodies and Flow Cytometry Analysis

The following mAbs from BD Biosciences were used: anti-CD45 (30-F11), anti-CD8 (53-6.7), anti-CD4 (RM4-5), anti-CD62L (MEL-14), anti-CD90.1 (OX-7), anti-CD45.1 (A20), anti-CD45.2 (104), anti-CD25 (PC61 or 7D4), anti-ICOS (7E.17G9), anti-GITR (DTA-1), anti-CD103 (M290), anti-Helios (22F6), anti-CTLA-4 (UC10-4F10-11), anti-CD11b (M1/70), anti-CD11c (HL3), anti-CD19 (1D3), anti-IA/E (M5/114.15.2), anti-Ly6C (AL-21), anti-Ly6G (1A8). Anti-GFP antibody was purchased from Life Technologies. Anti-CD3 (145-2C11), anti-Foxp3 (FJK-16s), anti-CD44 (IM7), anti-Ki-67 (SOLA15), anti-Nrp1 (3DS304M), anti-NKp46 (29A1.4) and anti-F4/80 (BM8) were purchased from eBioscience, and Foxp3 staining was performed using the eBioscience kit and protocol. Cells were acquired on a BD LSRII and a BD Fortessa X20 cytometers and analyzed using FlowJo software.

### Cytokine Quantification

Serum cytokines were quantified using the mouse Th1/Th2/Th17 Cytokine CBA Kit (BD Biosciences) according to manufacturer's procedure. Datas were analyzed using FCAP array software.

### Immunoglobulin and Autoantibody Quantification by ELISA

Ninety-six-well flat plates were coated with either salmon sperm DNA (Sigma) or with goat anti-mouse IgM, IgA, IgE, IgG1, IgG2b (Southern Biotech). After washes, they were saturated with BSA and first incubated with mice sera, then with biotinylated goat anti-mouse IgG (Southern Biotech) or goat anti-mouse IgM, IgA, IgE, IgG1, IgG2b (Southern Biotech). A streptavidin-horseradish conjugate (Sigma) was added followed by the addition of TMB (eBioscience). The reaction was stopped with HCl (1N) and revealed with an ELISA plate reader DTX880 Multimode Detector (Beckman Coulter).

### Treg and Tconv Cell Purification

Treg were purified after enrichment of CD25^+^ cells using biotinylated anti-CD25 mAb (7D4) and anti-biotin microbeads (Miltenyi Biotec), followed by CD4 staining (RM4.5) and cell sorting of CD4^+^ Foxp3/YFP^+^ cells or CD4+ Foxp3/GFP^+^ using the BD FACSAria II. Tconv cells were purified after enrichment of CD25^−^ cells using biotinylated anti-CD25 mAb (7D4) or of CD8^−^CD19^−^CD11b^−^ cells using biotinylated anti-CD8 (53-6.7), CD19 (1D3), and CD11b (M1/70) mAbs and anti-biotin microbeads (Miltenyi Biotec), followed by CD4 staining (RM4.5), and cell sorting of CD4^+^ Foxp3/YFP^−^ cells or CD4+ Foxp3/GFP^−^ using the BD FACSAria II.

### Cell Cultures

Purified Treg (CD4^+^YFP^+^, 25 × 10^3^ cells/well) were cultured with or without whole splenocyte from CD3KO mice (7.5 × 10^4^ cells/well), anti-CD3 mAb (0,05 μg/ml, BioXcell), TNF (50 ng/ml, Protein Service Facility, VIB, Belgium) and IL-2 (10 ng/ml, Peproteck) in a 96-well round plate in RPMI 1640–10% FCS. For suppression assays, after labeling with CellTrace Violet Proliferation Kit (Life technologies), Tconv cells (CD4^+^YFP^−^, 2.5 × 10^4^ cells/well) were co-cultures with various Treg (CD4^+^YFP^+^) numbers and stimulated by splenocytes from CD3 KO mice (7.5 × 10^4^ cells/well) and soluble anti-CD3 (0.05 μg/ml 2C11, BioXCell) in RPMI 1640–10% FCS.

### Colitis

Tconv cells (CD4^+^GFP^−^, 1 × 10^5^ cells) and Tregs (CD4^+^YFP^+^, 2 × 10^4^ cells) were injected intravenously into sex-matched RAG2^−/−^ mice. The clinical evaluation was performed three times a week by measuring body weight. Colitis was scored on tissue sections as described previously (56).

### T-Cell Adoptive Transfer

CD3 KO mice were co-transferred with Treg (CD4^+^YFP^+^, 1 × 10^5^ each) purified from age and sex-matched CD45.1/2 *Foxp3*^*Cre*/+^ and CD45.2/2 *Foxp3*^*Cre*/+^ *Rela*^*lox*^ mice and Tconv cells (CD4^+^GFP^−^, 8 × 10^5^) purified from CD90.1 *Foxp3*^*GFP*^ mice.

### Electrophoretic Mobility Shift Assays (EMSA) Combined With Supershit Assays

Nuclear extracts were prepared and analyzed for DNA binding activity using the HIV-LTR tandem κB oligonucleotide as κB probe (57). For supershift assays, nuclear extracts were incubated with specific antibodies for 30 min on ice before incubation with the labeled probe.

### Gene-Expression Profiling and Analysis

Tregs (1,000) were double-sorted into TRIzol (Invitrogen). Subsequent sample processing was followed by Ultra-low input RNAseq protocol as described (58). Normalized data were analyzed with Multiplot Studio, GSEA and Gene-e modules in Genepattern. For signature enrichment analysis, each signature was curated from published datasets and computed by comparison between two conditions (e.g., WT vs. KO). Data were downloaded from GEO and only the ones containing replicates were used. To reduce noise, genes with a coefficient of variation between biological replicates > 0.6 in either comparison groups were selected. Up- and down-regulated transcripts were defined as having a fold change in gene expression > 1.5 or <2/3 and a *t*-test *p*-value < 0.05. A signature score for each single cell was computed by summing the counts for the upregulated genes and subtracting the counts for the downregulated genes. Z scores were plotted in the heat map (Zemmour_Code/Zemmour_Code.Rmd: ^**^Treg signatures and single cell score^**^).

### Statistical Analysis

Statistical analyses were performed using GraphPad Prism Software. Statistical significance was determined using a log-rank (Mantel- Cox) test for the mouse survival data. For all the other statistical analysis, the two-tailed unpaired non-parametric Mann–Whitney *U*-test was used for data not following a normal distribution and the *t-*test was used for data following a normal distribution. ^*^p < 0.05, ^**^p < 0.01, ^***^p < 0.001, ^****^p < 0.0001. Means ± SEM were used throughout the figures.

## Data Availability Statement

The raw data supporting the conclusions of this manuscript will be made available by the authors, without undue reservation, to any qualified researcher.

## Ethics Statement

All experimental protocols were approved by the local ethics committee Comité d'éthique en expérimentation animal Charles Darwin N°5 under the number 02811.03 and are in compliance with European Union guidelines.

## Author Contributions

BS and ER designed the research. ER performed almost all the experiments and analyzed the data. ML, RV, JD, SG, and AR helped ER on some experiments. H-KK and CB performed and analyzed the RNA-seq data. DC and VB performed and analyzed the western blot and EMSA. BS and ER wrote the manuscript using comments from all authors.

### Conflict of Interest

The authors declare that the research was conducted in the absence of any commercial or financial relationships that could be construed as a potential conflict of interest.

## References

[1] Sakaguchi S Ono M Setoguchi R Yagi H Hori S Fehervari Z. Foxp3+CD25+CD4+ natural regulatory T cells in dominant self-tolerance and autoimmune disease. Immunol Rev. (2006) 212:8–27. 10.1111/j.0105-2896.2006.00427.x 16903903

[2] Isomura I Palmer S Grumont RJ Bunting K Hoyne G Wilkinson N. C-Rel is required for the development of Thymic Foxp3+ CD4 regulatory T cells. J Exp Med. (2009) 206:3001–14. 10.1084/jem.20091411 19995950 PMC2806473

[3] Long M Park S-G Strickland I Hayden MS Ghosh S. Nuclear factor-κB modulates regulatory T cell development by directly regulating expression of Foxp3 transcription factor. Immunity. (2009) 31:921–31. 10.1016/j.immuni.2009.09.022 20064449

[4] Wu Y Borde M Heissmeyer V Feuerer M Lapan AD Stroud JC. FOXP3 controls regulatory T cell function through cooperation with NFAT. Cell. (2006) 126:375–87. 10.1016/j.cell.2006.05.042 16873067

[5] Ono M Yaguchi H Ohkura N Kitabayashi I Nagamura Y Nomura T. Foxp3 controls regulatory T-cell function by interacting with AML1/Runx1. Nature. (2007) 446:685–9. 10.1038/nature05673 17377532

[6] Pan F Yu H Dang EV Barbi J Pan X Grosso JF. Eos mediates Foxp3-dependent gene silencing in CD4^+^ regulatory T cells. Science. (2009) 325:1142–6. 10.1126/science.1176077 19696312 PMC2859703

[7] Roychoudhuri R Hirahara K Mousavi K Clever D Klebanoff CA Bonelli M BACH2 represses effector programs to stabilize Treg-mediated immune homeostasis. Nature. (2013) 498:506–10. 10.1038/nature12199 23728300 PMC3710737

[8] Chaudhry A Rudra D Treuting P Samstein RM Liang Y Kas A. CD4^+^ regulatory T cells control T_H_17 responses in a Stat3-dependent manner. Science. (2009) 326:986–91. 10.1126/science.1172702 19797626 PMC4408196

[9] Koch MA Tucker-Heard G Perdue NR Killebrew JR Urdahl KB Campbell DJ. The transcription factor T-bet controls regulatory T cell homeostasis and function during type 1 inflammation. Nat Immunol. (2009) 10:595–602. 10.1038/ni.1731 19412181 PMC2712126

[10] Zheng Y Chaudhry A Kas A deRoos P Kim JM Chu TT Regulatory T-cell suppressor program Co-Opts transcription factor IRF4 to control TH2 responses. Nature. (2009) 458:351–6. 10.1038/nature07674 19182775 PMC2864791

[11] Cretney E Xin A Shi W Minnich M Masson F Miasari M. The transcription factors Blimp-1 and IRF4 jointly control the differentiation and function of effector regulatory T cells. Nat Immunol. (2011) 12:304–11. 10.1038/ni.2006 21378976

[12] Linterman MA Pierson W Lee SK Kallies A Kawamoto S Rayner TF. Foxp3+ follicular regulatory T cells control the germinal center response. Nat Med. (2011) 17:975–82. 10.1038/nm.2425 21785433 PMC3182542

[13] Cipolletta D Feuerer M Li A Kamei N Lee J Shoelson SE. PPAR-γ is a major driver of the accumulation and phenotype of adipose tissue Treg cells. Nature. (2012) 486:549–53. 10.1038/nature11132 22722857 PMC3387339

[14] Dias S D'Amico A Cretney E Liao Y Tellier J Bruggeman C. Effector regulatory T cell differentiation and immune homeostasis depend on the transcription factor Myb. Immunity. (2017) 46:78–91. 10.1016/j.immuni.2016.12.017 28099866

[15] Wohlfert EA Grainger JR Bouladoux N Konkel JE Oldenhove G Ribeiro CH. GATA3 controls Foxp3^+^ regulatory T cell fate during inflammation in mice. J Clin Invest. (2011) 121:4503–15. 10.1172/JCI57456 21965331 PMC3204837

[16] Feng Y Arvey A Chinen T van der Veeken J Gasteiger G Rudensky AY. Control of the inheritance of regulatory T cell identity by a cis element in the Foxp3 locus. Cell. (2014) 158:749–63. 10.1016/j.cell.2014.07.031 25126783 PMC4151558

[17] Liu Y Wang L Han R Beier UH Akimova T Bhatti T. Two histone/protein acetyltransferases, CBP and P300, are indispensable for Foxp3+ T-regulatory cell development and function. Mol Cell Biol. (2014) 34:3993–4007. 10.1128/MCB.00919-14 25154413 PMC4386456

[18] DuPage M Chopra G Quiros J Rosenthal WL Morar MM Holohan D. The chromatin-modifying enzyme Ezh2 is critical for the maintenance of regulatory T cell identity after activation. Immunity. (2015) 42:227–38. 10.1016/j.immuni.2015.01.007 25680271 PMC4347854

[19] Yang R Qu C Zhou Y Konkel JE Shi S Liu Y. Hydrogen sulfide promotes Tet1- and Tet2-mediated Foxp3 demethylation to drive regulatory T cell differentiation and maintain immune homeostasis. Immunity. (2015) 43:251–63. 10.1016/j.immuni.2015.07.017 26275994 PMC4731232

[20] Garg G Muschaweckh A Moreno H Vasanthakumar A Floess S Lepennetier G. Blimp1 prevents methylation of Foxp3 and loss of regulatory T cell identity at sites of inflammation. Cell Rep. (2019) 26:1854–68.e5. 10.1016/j.celrep.2019.01.070 30759395 PMC6389594

[21] Bettelli E Dastrange M Oukka M Foxp3 interacts with nuclear factor of activated T cells and NF-KB to repress cytokine gene expression and effector functions of T helper cells. Proc Natl Acad Sci USA. (2005) 102:5138–43. 10.1073/pnas.0501675102 15790681 PMC555574

[22] Ruan Q Kameswaran V Tone Y Li L Liou H-C Greene MI. Development of Foxp3+ regulatory T cells is driven by the C-Rel enhanceosome. Immunity. (2009) 31:932–40. 10.1016/j.immuni.2009.10.006 20064450 PMC2807990

[23] Chang J-H Xiao Y Hu H Jin J Yu J Zhou X. Ubc13 maintains the suppressive function of regulatory T cells and prevents their conversion into effector-like T cells. Nat Immunol. (2012) 13:481–90. 10.1038/ni.2267 22484734 PMC3361639

[24] Heuser C Gotot J Piotrowski EC Philipp M-S Courrèges CJF Otte MS. Prolonged IKKβ inhibition improves ongoing CTL antitumor responses by incapacitating regulatory T cells. Cell Rep. (2017) 21:578–86. 10.1016/j.celrep.2017.09.082 29045828

[25] Chen X Willette-Brown J Wu X Hu Y Howard OMZ Hu Y. IKKα is required for the homeostasis of regulatory T cells and for the expansion of both regulatory and effector CD4 T cells. FASEB J. (2015) 29:443–54. 10.1096/fj.14-259564 25376833 PMC4314223

[26] Oh H Ghosh S NF-KB: roles and regulation in different CD4+ T-cell subsets. Immunol Rev. (2013) 252:41–51. 10.1111/imr.12033 23405894 PMC3576882

[27] Kwon HK Chen HM Mathis D Benoist C. Different molecular complexes that mediate transcriptional induction and repression by FoxP3. Nat Immunol. (2017) 18:ni.3835. 10.1038/ni.3835 28892470 PMC5679728

[28] Messina N Fulford T O'Reilly L Loh WX Motyer JM Ellis D. The NF-κB transcription factor RelA is required for the tolerogenic function of Foxp3+ regulatory T cells. J Autoimmun. (2016) 70:52–62. 10.1016/j.jaut.2016.03.017 27068879

[29] Vasanthakumar A Liao Y Teh P Pascutti MF Oja AE Garnham AL. The TNF receptor superfamily-NF-κB axis is critical to maintain effector regulatory T cells in lymphoid and non-lymphoid tissues. Cell Rep. (2017) 20:2906–20. 10.1016/j.celrep.2017.08.068 28889989

[30] Oh H Grinberg-Bleyer Y Liao W Maloney D Wang P Wu Z. An NF-κB transcription-factor-dependent lineage-specific transcriptional program promotes regulatory T cell identity and function. Immunity. (2017) 47:450–465.e5. 10.1016/j.immuni.2017.08.010 28889947 PMC5679261

[31] Algül H Treiber M Lesina M Nakhai H Saur D Geisler F. Pancreas-specific RelA/P65 truncation increases susceptibility of acini to inflammation-associated cell death following cerulein pancreatitis. J Clin Invest. (2007) 117:1490–501. 10.1172/JCI29882 17525802 PMC1868784

[32] Kaech SM Hemby S Kersh E Ahmed R. Molecular and functional profiling of memory CD8 T cell differentiation. Cell. (2002) 111:837–51. 10.1016/S0092-8674(02)01139-X 12526810

[33] Hill JA Feuerer M Tash K Haxhinasto S Perez J Melamed R. Foxp3 transcription-factor-dependent and -independent regulation of the regulatory T cell transcriptional signature. Immunity. (2007) 27:786–800. 10.1016/j.immuni.2007.09.010 18024188

[34] Zemmour D Zilionis R Kiner E Klein AM Mathis D Benoist C Single-cell gene expression reveals a landscape of regulatory T cell phenotypes shaped by the TCR. Nat Immunol. (2018) 19:291–301. 10.1038/s41590-018-0051-0 29434354 PMC6069633

[35] Zhou X Bailey-Bucktrout SL Jeker LT Penaranda C Martínez-Llordella M Ashby M. Instability of the transcription factor Foxp3 leads to the generation of pathogenic memory T cells *in vivo*. Nat Immunol. (2009) 10:1000–7. 10.1038/ni.1774 19633673 PMC2729804

[36] Hilliard BA Mason N Xu L Sun J Lamhamedi-Cherradi S-E Liou H-C. Critical roles of C-Rel in autoimmune inflammation and helper T cell differentiation. J Clin Invest. (2002) 110:843–50. 10.1172/JCI15254 12235116 PMC151124

[37] Ruan Q Kameswaran V Zhang Y Zheng S Sun J Wang J. The Th17 immune response is controlled by the Rel–RORγ-RORγT transcriptional axis. J Exp Med. (2011) 208:2321–33. 10.1084/jem.20110462 22006976 PMC3201209

[38] Sharma R Sung SJ Fu SM Ju ST. Regulation of multi-organ inflammation in the regulatory T cell-deficient scurfy mice. J. Biomed. Sci. (2009) 16:20. 10.1186/1423-0127-16-20 19272184 PMC2653523

[39] Hadaschik EN Wei X Leiss H Heckmann B Niederreiter B Steiner G. Regulatory T cell-deficient scurfy mice develop systemic autoimmune features resembling lupus-like disease. Arthritis Res Ther. (2015) 17:35. 10.1186/s13075-015-0538-0 25890083 PMC4391674

[40] Sather BD Treuting P Perdue N Miazgowicz M Fontenot JD Rudensky AY. Altering the distribution of Foxp3+ regulatory T cells results in tissue-specific inflammatory disease. J Exp Med. (2007) 204:1335–47. 10.1084/jem.20070081 17548521 PMC2118615

[41] Kitamura K Farber JM Kelsall BL CCR6 marks regulatory T cells as a colon-tropic, interleukin-10-producing phenotype. J Immunol. (2010) 185:3295–304. 10.4049/jimmunol.1001156 20720211 PMC3932491

[42] Ye J Qiu J Bostick JW Ueda A Schjerven H Li S. The Aryl hydrocarbon receptor preferentially marks and promotes gut regulatory T cells. Cell Rep. (2017) 21:2277–90. 10.1016/j.celrep.2017.10.114 29166616 PMC5880207

[43] van der Veeken J Gonzalez AJ Cho H Arvey A Hemmers S Leslie CS. Memory of inflammation in regulatory T cells. Cell. (2016) 166:977–90. 10.1016/j.cell.2016.07.006 27499023 PMC4996371

[44] Polansky JK Kretschmer K Freyer J Floess S Garbe A Baron U. DNA methylation controls Foxp3 gene expression. Eur J Immunol. (2008) 38:1654–63. 10.1002/eji.200838105 18493985

[45] Mukherjee SP Behar M Birnbaum HA Hoffmann A Wright PE Ghosh G. Analysis of the RelA:CBP/p300 interaction reveals its involvement in NF-κB-driven transcription. PLoS Biol. (2013) 11:e1001647. 10.1371/journal.pbio.1001647 24019758 PMC3760798

[46] van Loosdregt J Coffer PJ. Post-translational modification networks regulating FOXP3 function. Trends Immunol. (2014) 35:368–78. 10.1016/j.it.2014.06.005 25047417

[47] Soligo M Camperio C Caristi S Scott,à C Del Porto P Costanzo A CD28 costimulation regulates FOXP3 in a RelA/NF-KB-dependent mechanism. Eur J Immunol. (2011) 41:503–13. 10.1002/eji.201040712 21268019

[48] Camperio C Caristi S Fanelli G Soligo M Del Porto P Piccolella E. Forkhead transcription factor FOXP3 upregulates CD25 expression through cooperation with RelA/NF-κB. PLoS ONE. (2012) 7:e48303. 10.1371/journal.pone.0048303 23144749 PMC3483148

[49] Vallabhapurapu S Karin M. Regulation and function of NF-κB transcription factors in the immune system. Annu Rev Immunol. (2009) 27:693–733. 10.1146/annurev.immunol.021908.132641 19302050

[50] Gerondakis S Siebenlist U. Roles of the NF-κB pathway in lymphocyte development and function. Cold Spring Harb Perspect Biol. (2010) 2:a000182. 10.1101/cshperspect.a000182 20452952 PMC2857169

[51] Hayden MS Ghosh S. NF-κB in immunobiology. Cell Res. (2011) 21:223–44. 10.1038/cr.2011.13 21243012 PMC3193440

[52] Li-Weber M Giaisi M Baumann S Pálfi K Krammer PH NF-κB synergizes with NF-AT and NF-IL6 in activation of the IL-4 gene in T cells. Eur J Immunol. (2004) 34:1111–8. 10.1002/eji.200324687 15048722

[53] Balasubramani A Shibata Y Crawford GE Baldwin AS Hatton RD Weaver CT. Modular utilization of distal cis-regulatory elements controls Ifng gene expression in T cells activated by distinct stimuli. Immunity. (2010) 33:35–47. 10.1016/j.immuni.2010.07.004 20643337 PMC2994316

[54] Rubtsov YP Rasmussen JP Chi EY Fontenot J Castelli L Ye X. Regulatory T cell-derived interleukin-10 limits inflammation at environmental interfaces. Immunity. (2008) 28:546–58. 10.1016/j.immuni.2008.02.017 18387831

[55] Wang Y Kissenpfennig A Mingueneau M Richelme S Perrin P Chevrier S. Th2 lymphoproliferative disorder of *Lat^Y136F^* mutant mice unfolds independently of TCR-MHC engagement and is insensitive to the action of Foxp3^+^ regulatory T cells. J Immunol. (2008) 180:1565–75. 10.4049/jimmunol.180.3.1565 18209052

[56] Martin B Auffray C Delpoux A Pommier A Durand A Charvet C. Highly self-reactive naive CD4 T cells are prone to differentiate into regulatory T cells. Nat Commun. (2013) 4:ncomms3209. 10.1038/ncomms3209 23900386

[57] Jacque E Billot K Authier H Bordereaux D Baud V. RelB inhibits cell proliferation and tumor growth through P53 transcriptional activation. Oncogene. (2013) 32:2661–9. 10.1038/onc.2012.282 22777360

[58] Zemmour D Pratama A Loughhead SM Mathis D Benoist C. Flicr, a long noncoding RNA, modulates Foxp3 expression and autoimmunity. Proc Natl Acad Sci USA. (2017) 114:E3472–80. 10.1073/pnas.1700946114 28396406 PMC5410798

