# Supplementary material for: The NF-κB RelA Transcription Factor Is Critical for Regulatory T Cell Activation and Stability
Source: Front Immunol. 2019 Oct 30;10:2487. doi: 10.3389/fimmu.2019.02487 (PMC6842949; doi:10.3389/fimmu.2019.02487)
Supplement: Supplementary file 1 [file Data_Sheet_1.docx]

**Supplementary Figure 1. Systemic activation of CD8^+^ and CD4^+^ conventional T cells in *Foxp3^Cre^ Rela^lox^* mice.** (A) Representative dot plots and proportion of ICOS^+^ among CD8^+^ and CD4^+^ conventional T cells in different tissues (spl=spleen) of *Foxp3^Cre^* (*Cre*) and *Foxp3^Cre^ Rela^lox^* (*Cre Rela^lox^*). (B) Proportion of CD44^hi^CD62L^low^, Ki67^+^ and ICOS^+^ among CD8^+^ and CD4^+^ conventional T cells in different tissues (liv=liver, SI= small intestine) of *Foxp3^Cre^* and *Foxp3^Cre^ Rela^lox^*. Bars represent the means pooled from independent experiments in 12 week-old mice. Error bars represent SEM. For mouse and experiment numbers, see Supplementary Table 1. The two-tailed unpaired nonparametric Mann–Whitney *U* test was used for data not following a normal distribution and the *t-*test was used for data following a normal distribution. **p*<0.05, ***p*<0.01, ****p*<0.001, *****p*<0.0001.

**Supplementary Figure 2. Increased numbers of myeloid cells in *Foxp3^Cre^ Rela^lox^* mice.** (A) Gating strategy of myeloid and NK cells. (B, C) Number of CD11b^+^ (B) and of neutrophils, eosinophils, inflammatory monocytes and monocytes (C) in the indicated organs (spl=spleen) of 12 week-old *Foxp3^Cre^* (*Cre*) and *Foxp3^Cre^ Rela^lox^* (*Cre Rela^lox^*) mice. Bars represent the means pooled from independent experiments in 12 week-old mice. Error bars represent SEM. For mouse and experiment numbers, see Supplementary Table 1. The two-tailed unpaired nonparametric Mann–Whitney *U* test was used for data not following a normal distribution and the *t-*test was used for data following a normal distribution. **p*<0.05, ***p*<0.01, ****p*<0.001, *****p*<0.0001.

**Supplementary Figure 3. Homeostasis and phenotype of T cells in 5 week-old *Foxp3^Cre^ Rela^lox^* mice.** Number of CD45^+^ (A) and proportion of CD44^hi^CD62L^low^, ICOS^+^ and Ki67^+^ among CD8^+^ and CD4^+^ conventional T cells (B) in different tissues (spl=spleen, liv=liver, SI= small intestine) of *Foxp3^Cre^* (*Cre*) and *Foxp3^Cre^ Rela^lox^* (*Cre Rela^lox^*). Each dot represents a mouse and lines show the means pooled from independent experiments in 12 week-old mice. For mouse and experiment numbers, see Supplementary Table 1. The two-tailed unpaired nonparametric Mann–Whitney *U* test was used for data not following a normal distribution and the *t-*test was used for data following a normal distribution. **p*<0.05, ***p*<0.01.

**Supplementary Figure 4.** **Treg number in 12 and 5 week-old *Foxp3^Cre^ Rela^lox^* mice.** Number of Tregs in different tissues (thy=thymus, spl=spleen, liv=liver, SI= small intestine) in 12 (A) and 5 (B) week-old *Foxp3^Cre^* (*Cre*) and *Foxp3^Cre^ Rela^lox^* (*Cre Rela^lox^*) mice. Each dot represents a mouse and lines show the means pooled from independent experiments. For mouse and experiment numbers, see Supplementary Table 1. The two-tailed unpaired nonparametric Mann–Whitney *U* test was used for data not following a normal distribution and the *t-*test was used for data following a normal distribution. **p*<0.05, ***p*<0.01, ****p*<0.001, *****p*<0.0001.

**Supplementary Figure 5.** **Tconv survival and pTreg number in lymphopenic mice transferred with Tconv and RelA-deficient Tregs.** Tconv (CD90.1^+^) and Tregs (CD90.1^-^) were injected in RAG KO mice to induce colitis as described in figure 4E-G. (A) Representative gating strategy. (B) Whole number of recovered Tconv (CD90.1^+^) and of pTregs (CD90.1^+^Foxp3^+^) in the mLN and colon of mice injected with Tconv only, Tconv and WT Tregs or Tconv and RelA KO Tregs analyzed 6 weeks after transfer. Each dot represents a mouse and lines show the means pooled from independent experiments. For mouse and experiment numbers, see Supplementary Table 1.

**Supplementary Figure 6.** **ICOS, CTLA4, Nrp1 and Helios expression in RelA-deficient Tregs at steady state.** Analyses in the indicated organs (spl=spleen, liv=liver) of 8 week-old *Foxp3^Cre/wt^* (*Cre/wt* – grey bars) and *Foxp3^Cre/wt^ Rela^lox^* (*Cre/wt Rela^lox^* – green bars) mice. Representative dot plots from sdLN and proportions of (A) ICOS^+^, (B) CTLA4^+^, (C) Nrp1^+^ and (D) Helios^+^ among CRE^+^ Tregs. Bars show the means of pooled independent experiments and error bars represent SEM. For mouse and experiment numbers, see Supplementary Table 1. The two-tailed unpaired nonparametric Mann–Whitney *U* test was used. **p*<0.05.

| **Figure** | **Total number of mice** | **Number of independent experiments** |
| --- | --- | --- |
| 1A | 4 per group | 2 |
| 1C, D, E | 9 *Cre*, 20 *Cre Rela lox* | 1 |
| 1F | 20 | 4 |
| 1G | 6 to 8 per group | 2 |
| 1H | 4 per group | 2 |
| 2A | 18 to 29 per group | 8 |
| 2B | 10 to 24 per group | 3 (Bcells), 8 (CD4, CD8) |
| 2C,D | 3 to 12 per group | 2 (skin, Ki67 in colon), 3 (all the other ones) |
| 2E,F | 7 per group | 2 |
| 2G | 15 *Cre*, 6 4-12 week-old *Cre Rela lox*, 9 12-14 week-old *Cre Rela lox* | 2 |
| 3A | 11 to 24 per group | 3 (Thy), 8 (all the other ones) |
| 3B | 7 to 12 per group | 3 (SI, colon, skin), 4 (all the other ones) |
| 3C | 6 to 12 per group | 3 |
| 3D | - | 4 |
| 3E | 19 to 20 per group | 4 |
| 3F | 4 per group | 1 |
| 3G | 5 to 7 per group | 2 |
| 4B | 4 per group | 2 |
| 4C,D,E | 6 to 10 per group | 3 |
| 4F | 5 per group | 2 |
| 5A | 8 to 13 per group | 3 |
| 5B-E | 4 to 10 per group | 3 |
| 6 | 6 per group | 2 |
| 7B,C | 8 to 11 per group | 3 |
| 7D | 8 per group | 2 |
| 7E | 4 per group | 2 |
| Supp 1A,B | 3 to 16 per group | 2 (skin, ICOS in colon), 3 (all the other ones) |
| Supp 2B,C | 8 to 10 per group | 3 |
| Supp 3A | 8 to 12 per group | 3 (SI, colon, skin), 4 (all the other ones) |
| Supp 3B | 2 to 8 per group | 1 (CD44hi CD62L, Ki67 in lung), 2 (all the other ones) |
| Supp 4A | 11 to 24 per group | 3 (Thy), 8 (all the other ones) |
| Supp 4B | 7 to 12 per group | 3 (SI, colon, skin), 4 (all the other ones) |
| Supp 5B | 5 to 7 per group | 2 |
| Supp 6 | 6 to 10 per group | 2 (Nrp1, Helios), 3 (ICOS, CTLA4) |

**Supplementary Table 1**. Mouse and experiment numbers of each panel
